# A multi-center study on the predictors of different subtypes of hemorrhagic transformation of brain infarction after thrombolysis in atrial fibrillation patients presented with embolic stroke

**DOI:** 10.1038/s41598-025-97968-3

**Published:** 2025-05-05

**Authors:** Sherihan Rezk Ahmed, Mohamed G. Zeinhom, Ahmed Ahmed Mohamed Kamal Ebied, Islam Fathallah Mohamed Kamel, Mohamed Ahmed Almoataz, Ahmed Mohamed Ali Daabis, Ahmed Zaki Omar Akl, Emad Labib Abdelhamid Mahmoud, Adnan Ghazi Alkhalefeh, Shady G. Ouf, Suaad Abdallah Ali Mosbah, Igbal Mubarak Ismail Sirag, Mohamed Abouelnaga, Mohamed Fouad Elsayed Khalil

**Affiliations:** 1 Neurology Department, Faculty of Medicine, Kafr El-Sheikh University, Elgeish Street, Kafr El-Sheikh, Egypt; 2 Neurology Department, Faculty of Medicine, Zagazig University, 2 Elgeish Et, Zagazig, Egypt; 3 Neurology Department, Faculty of Medicine, Tanta University, ELbahr St., Tanta, Egypt; 4 Neurology Department, Saudi German Hospital, Sharjah, United Arab Emirates; 5 Neurology Department, Burjeel Royal Hospital, Al Ain, United Arab Emirates; 6 Neurology Department, Faculty of Medicine, Ain Shams University, Al Khalifa Elmamon St., Cairo, Egypt; 7 Cardiology Department, National Heart Institute, Imbaba District, Cairo, Egypt; 8 Cardiology Department, NMC Royal Hospital-MBZ, Abu Dhabi, United Arab Emirates; 9 Cardiology Department, Phoenix Hospital, Abu Dhabi, United Arab Emirates; 10 Cardiology Department, Faculty of Medicine, Zagazig University, 2 Elgeish Et, Zagazig, Egypt; 11 Internal Medicine Department, NMC Royal Hospital-MBZ, Abu Dhabi, United Arab Emirates; 12 Neurology Department, Faculty of Medicine, Alexandria University, Elgomhorea St., Alexandria, Egypt; 13 Neurology Department, NMC Royal Hospital Mohamed Bin Zayed, Abu Dhabi, United Arab Emirates

**Keywords:** Alteplase, Hemorrhagic infarction, Egypt, United Arab Emirates, Embolic stroke, Neuroscience, Diseases of the nervous system, Neuro-vascular interactions

## Abstract

Embolic stroke is connected to a higher risk of hemorrhagic transformation (HT), functional disability, and mortality. Although, AF and HT are not one entity; no such study evaluated the factors, including AF types and treatment, which could predict the different types of HT in AF patients presenting with embolic stroke and administered alteplase. We aimed to assess the predictors of HT in general and predictors of different ECASS-based subtypes of post-alteplase HT in AF patients who experienced first-ever embolic ischemic stroke. Our study included 716 AF patients who presented with acute embolic stroke and received the full recommended dose of alteplase. The study comprised six parallel groups. The first group consisted of 509 patients who did not experience haemorrhagic transformation. The second group comprised 207 patients who had any HT. The third group comprised 87 patients with haemorrhagic infarction (HI)1. The fourth group comprised 62 patients with HI2. The fifth group comprised 33 patients with parenchymal hematoma (PH) 1, and the sixth group comprised 25 patients with PH 2. We evaluated the ability of different baseline characters and risk factors to predict the occurrence of HT in general and the predictors of occurrence of different ECASS-based HT subtypes. HT was detected in 207 patients (28.9%), older age, higher NIHSS, sustained AF, warfarin use, and higher HAS-BLED score were independent predictors of all ECASS-based subtypes of hemorrhagic transformation; moreover, anterior-circulation stroke was an independent predictor of PH 1 and PH 2. In atrial fibrillation patients presented with first-ever embolic stroke and received alteplase in Egypt and the United Arab Emirates, older age, higher NIHSS, sustained AF, warfarin use, and higher HAS-BLED score were independent predictors of all ECASS-based subtypes of haemorrhagic infarction; in addition, anterior-circulation stroke was an independent predictor of PH 1 and PH 2.

*Trial registration* (clinicaltrials.gov NCT06653946), retrospectively registered on 23/10/2024.

## Introduction

Stroke is the second leading cause of mortality on a global scale. Developing countries bear a disproportionate burden of stroke, representing 66% of the total stroke cases worldwide^1^.

The only approved medical treatment for ischemic stroke is alteplase; on the other hand, about 5% of patients who administered alteplase suffered from hemorrhagic transformation of the infarction, which worsened the outcome and increased post-stroke functional dependence^2,3^.

Atrial fibrillation (AF) is considered the most common cardiac arrhythmia in adults; it is one of the main risk factors associated with ischemic stroke, as about a quarter of ischemic stroke patients experience AF; moreover, embolic stroke is connected to a higher risk of hemorrhagic transformation, functional disability, and mortality^4,5^.

Many studies showed that embolic stroke caused by AF was associated with an increased risk of HT, functional disability, and mortality^6–8^, and other studies showed that using warfarin was linked to higher risks of intracranial hemorrhage in atrial fibrillation patients^9,10^; all these studies did not evaluate the different predictors of ECASS-based subtypes of HT in AF patients presenting with embolic stroke and administered alteplase, in addition, there was no such study that evaluated the ability of AF types and treatment to predict the HT different types in AF patients presenting with embolic stroke and received alteplase.

We aimed to assess the ability of different factors, including AF types and treatment, to predict the ECASS-based different subtypes of post-alteplase HT in atrial fibrillation patients presenting with embolic stroke.

## Methods

### Study design

Our prospective observational study was conducted between November 2022 and November 2024 after we received ethical committee approval, and all of our methods were performed following the relevant guidelines, regulations and declaration of Helsinki.

### Setting

Our study consisted of 716 atrial fibrillation patients who presented with first-ever embolic stroke and treated with alteplase within four and a half hours; 237 patients were recruited from Kafr el-Sheikh hospitals, 236 from Al-Obour hospitals in Egypt, and 243 from NMC hospitals in Abu Dhabi, United Arab Emirates.

Before inclusion in our study, we got formal written informed consent from all eligible patients or their first order of kin.

### Participants

The study consisted of six parallel groups. The first group consisted of 509 patients who did not experience HT. The second group comprised 207 patients who had any HT. The third group comprised 87 patients who had HI 1. The fourth group comprised 62 patients who had HI 2. The fifth group comprised 33 patients with PH 1, and the sixth group comprised 25 patients with PH 2.

Before inclusion in our study, we got formal written informed consent from all eligible patients or their first order of kin.

### Inclusion criteria

Our study included AF patients aged 18 to 80 years who presented with their first-ever acute embolic ischemic stroke and were eligible to receive alteplase; patients on direct oral anticoagulants (DOAC) received the last dose of DOAC 48 h before stroke onset and PT less than 15 s, aPTT less than 40 s, while patients who used warfarin had INR less than 1.7. All of our patients received the recommended full dose of alteplase. There was no imaging evidence of intracranial hemorrhage^11^.

We diagnosed cardio-embolic strokes when the participant had major or minor risk factors of a potential cardiac source of embolus, such as mechanical cardiac valves, AF, mitral valve prolapse, aortic valve stenosis or calcification, and patent foramen ovale^12,13^.

We diagnosed clinical AF if we had a minimum of 30 s of cardiac rhythm, showing the absence of P waves and irregular RR intervals (when atrioventricular conduction is not impaired) in a conventional 12-lead ECG recording^14^.

We did not rule out patients who suffered from previous transient ischemic attack (TIA).

### Exclusion criteria

We did not include patients with contraindications to alteplase or did not receive the alteplase total dose, patients who experienced an intracranial tumor, and participants with a history of recurrent CNS disorders such as multiple sclerosis, epilepsy or a history of head trauma resulting in permanent neurological impairment.

We ruled out patients who experienced end-organ failure, myocardial infarction during the preceding six weeks, and patients who experienced malignant tumors.

We ruled out pregnant and lactating participants, those who experienced venous sinus thrombosis, and patients who experienced stroke following cardiac arrest or severe hypotension.

### Study procedures

We recorded the baseline characters and the data regarding patients’ risk factors such as age, sex, medical history of hypertension (HTN), ischemic heart disease (IHD), hyperlipidemia, diabetes mellitus, AF types and treatment, cigarette use, treatment history and the duration between symptom onset and treatment initiation.

We diagnosed ischemic stroke after taking a thorough history and performing a clinical examination and suitable brain imaging. On admission, all of our patients had CT of the brain and CT angiography (CTA) including the aortic arch through the circle of Willis before thrombolysis; the CT of the brain was performed on the 64-slice dual-source spiral CT scanner of Somatom definition by Siemens, and the supratentorial compartment scans were imaged with 5–8 mm contiguous sections and the brain stem and cerebellum scans were imaged with 3–5 mm slices. We obtained eighteen images for each series. After thrombolysis, all of our patients underwent an MRI brain on a 1.5 T (Siemens Essenza) MR system, stroke protocol: T1W, T2W, fluid attenuation inversion recovery imaging (FLAIR), diffusion-weighted imaging (DWI), T2 Echo Gradient*, *and MRA brain and neck time of flight (TOF) if CTA was contraindicated and in our study 29 (4.1%) patients underwent MRA (TOF) brain& neck instead of CTA as they had renal impairment; all patients underwent brain CT scan on the 64-slice dual-source spiral CT scanner of Somatom definition by Siemens after 24–36 h after thrombolysis to evaluate any hemorrhagic transformation^15^. Moreover, we diagnosed symptomatic hemorrhagic transformation when the patient’s NIHSS score increased by 4 points or more^16^.

All the patients underwent trans-esophageal echocardiography, 12-lead routine ECG 24, 24 h continuous cardiac rhythm monitoring, and a daily 12-lead routine ECG during their stay in the hospital, permanent AF patients had ECG showed AF at the time of enrollment and no evidence of sinus rhythm in the 6 months before enrollment in the study, while patients with paroxysmal or persistent AF may not have been in AF at the time of enrollment, but had ECG-documented AF on two separate occasions, at least two weeks apart, in the 6 months before inclusion in the study^17^.

We classified AF into two groups: paroxysmal AF and sustained AF. We defined sustained AF as the combination of persistent AF and permanent AF, as it is often challenging to differentiate persistent AF and permanent AF in daily clinical practice^18^.

Our patients received rate control therapy in the acute setting to control the heart rate and reduce symptoms such as Beta-blockers, diltiazem, verapamil, or digoxin^19^.

We employed the HAS-BLED (hypertension, abnormal renal/liver function, stroke, bleeding history or predisposition, labile INR [international normalized ratio], elderly, drugs/alcohol concomitantly) score as a measure of baseline bleeding risk, as the result of adding 1 point to hypertension when systolic blood pressure more than 160 mmHg, abnormal renal function added 1 point when there was chronic renal failure or renal transplantation and/or serum creatinine ≥ 200 µmol/L (> ~ 2.3 mg/dL), abnormal liver function added 1 point when there was liver cirrhosis or laboratory tests showed increased bilirubin more than twice normal value and/or increased liver enzymes more than three times than normal, previous history of stroke ;this was set to zero as all of our patients had first-ever stroke, bleeding history or tendency added 1 point, labile international normalized ratio (INR) added one point when the target therapeutic INR achieved only during less than 60% of time of using anticoagulant, old age (older than 65 years) added one point, and use of drugs and alcohol concomitantly (1 point for each)^20^.

We identified posterior-circulation stroke (PCS) when ischemic occlusion affected basilar, posterior cerebral or vertebral arteries. In contrast, Anterior-circulation stroke (ACS) was determined when ischemic occlusion affected the internal carotid, middle, or anterior cerebral arteries^21^.

We evaluated the blood pressure of all of our participants and identified acute hypertensive response when the systolic blood pressure (BP) ≥ 140 mm Hg or diastolic (BP) ≥ 90 mm Hg was detected on two recordings taken 5 min apart within 24 h of stroke symptom onset^22^, we recorded the renal and liver functions, coagulation profile, complete blood count, fasting, postprandial blood sugar, and HbA1C, and we identified diabetes if the fasting plasma glucose level was more than 126 mg/dl or casual plasma glucose was more than 200 mg/dl, or HbA1C was more than 6.5^23,24^. Admission hyperglycemia was diagnosed when admission blood glucose value was more than 140 mg/dL^16^. We evaluated the lipid profile on admission and diagnosed hyperlipidemia when blood cholesterol was more than 200 mg/dL; triglycerides were more than150mg/dL, LDL-cholesterol was more than 100 mg/dL and/or HDL-cholesterol was less than 40 mg/dL)^25^. We evaluated the temperature of our patients orally at admission and twice daily during hospitalization (Temp Plus II, Ivac, Giessen, Germany) and identified fever when at least one measurement was ≥ 38 °C (sub-febrile temperature 37.6–37.9 °C)^26^.

If needed, participants who suffered from fever underwent a chest radiograph, urine analysis, and cultures for sputum, stool, and blood cultures. We treated the fever with one gram of acetaminophen early after hospitalization and antibiotics according to culture and sensitivity tests^26^.

All of our participants received 0.9 mg/kg of alteplase up to a maximum dose of 90 mg intravenously within 4.5 h of the beginning of their clinical manifestations (10% bolus, 90% infusion in 1 h). Our patients continued treatment and rehabilitation in our stroke intensive or intermediate care units according to their clinical conditions^11^.

### Variables

#### Primary outcome

We evaluated the predictors all HT at 0 to 36 h after IV alteplase.

We identified hemorrhagic transformation according to the European Cooperative Acute Stroke Study (ECASS) when intracranial hemorrhage was detected in the post-treatment imaging after receiving alteplase and increase of NIHSS by 4 points from baseline or death and classified into HI 1, HI 2; PH 1, and PH 2^27^.

#### Secondary outcomes

We evaluated the predictors of each ECASS-based subtype of HT including HI 1, HI 2, PH 1, and PH 2 at 0 to 36 h after IV alteplase.

#### Study size

We calculated the power of our study using the Power Analysis and Sample Size System (PASS, V12), NCSS) given the two-sided significance level of 95%, alpha error of 5%, mean and standard deviation of non-HT age (59.3 ± 9.4), mean and standard deviation of HT age (73.8 ± 4.3), an effect size of 0.306 and the power of our study was 92.6%.

### Statistical analysis of the data

We used the IBM SPSS software package, version 20.0 (Armonk, NY: IBM Corp.), to analyze our data and base all efficacy analyses on the per-protocol analysis principle. Both the primary and secondary outcomes underwent separate statistical analyses. Depending on their distribution, as determined by the Shapiro–Wilk test, we described numerical data as means S.D. or median and interquartile range (IQR). We also reported categorical data using numbers and percentages. The Mann–Whitney U test was used to compare the irregularly distributed numerical data, while the Chi-square test was applied to investigate the association between the categorical variables. Alternatively, Fisher’s Exact or Monte Carlo correction test was used when more than 20% of the cells had an expected count of less than five. Univariate and multivariate logistic regression analyses evaluated the predictors of different HT subtypes. The significance of the obtained results was judged at the 5% level.

## Results

**Fig. 1:**
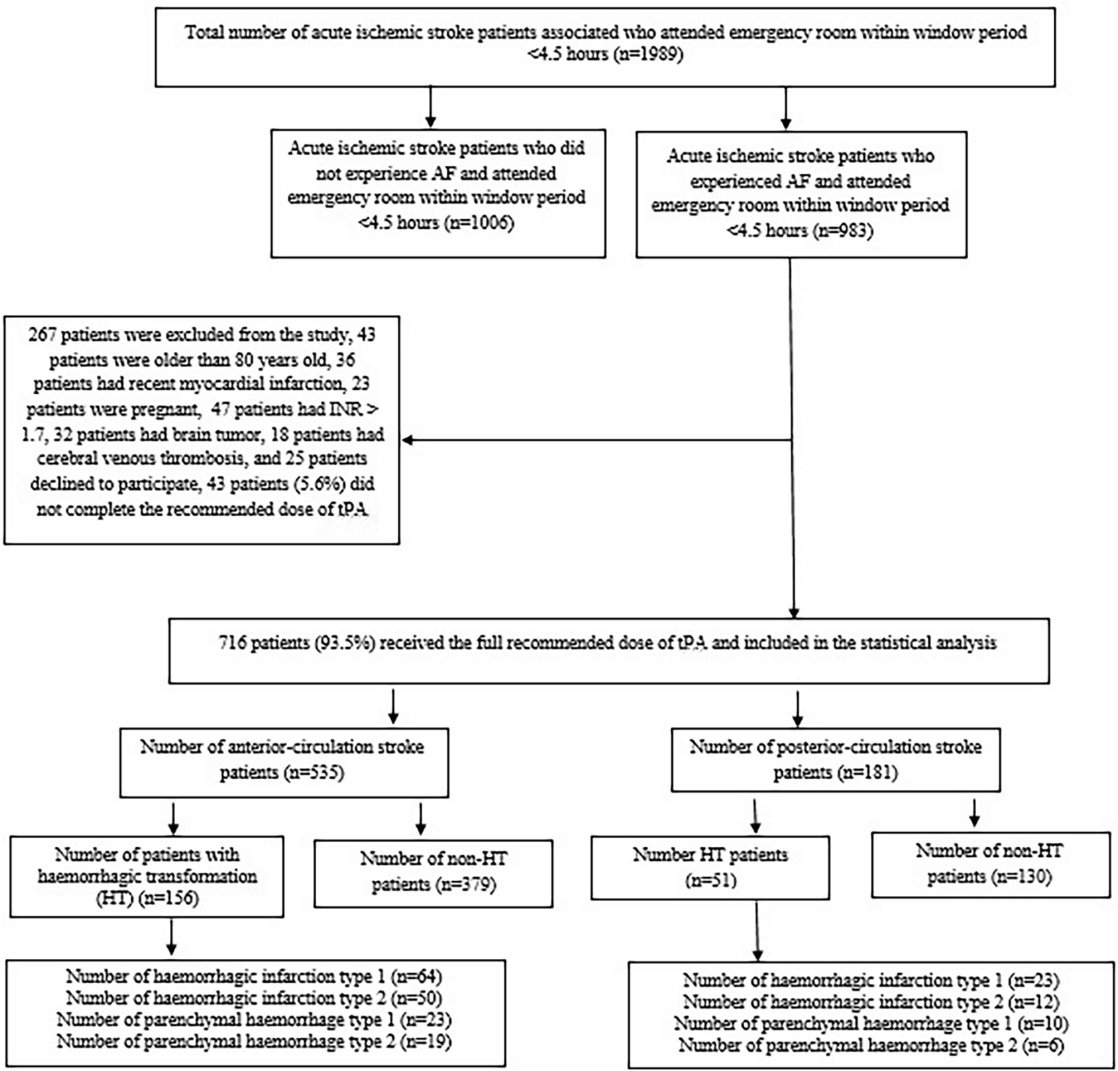
Study flow diagram.

Overall, we screened 1989 patients who came to the emergency with clinical manifestations of acute ischemic stroke within the therapeutic window of alteplase, of which 983 patients had ischemic stroke associated with AF. 267 patients were excluded from the study: 43 patients were older than 80 years old, 36 patients had recent myocardial infarction, 23 patients were pregnant, 47 patients had INR > 1.7, 32 patients had brain tumors, 18 patients had cerebral venous thrombosis, and 25 patients declined to participate, 43 patients (5.6%) did not complete the recommended dose of tPA, and 716 patients (271 females & 445 males) received the total recommended dose of alteplase. They were included in the per-protocol analysis; 207 (28.9%) had HT, 87 patients (12.2%) had HI 1, 62 patients (8.7%) had HI 2, 33 patients (4.6%) had PH 1, 25 patients (3.5%) had PH 2, as shown in Fig. 1.

Although patients with sustained AF are more likely to be anticoagulated before stroke than those with paroxysmal AF are, There were no significant differences between the sustained and paroxysmal AF groups regarding the rate of anticoagulant medications used in each group as follows: 55 patients in the sustained AF group and 23 patients in the paroxysmal AF group were warfarin users with *P*-value 0.31, 27 patients in the sustained AF group and 12 in the paroxysmal AF group were rivaroxaban users with *P*-value 0.66, 25 patients in the sustained AF group and 11 in the paroxysmal AF group were apixaban users with *P*-value 0.65, 12 patients in the sustained AF group and three in the paroxysmal AF group were Endoxaban users with *P*-value 0.24, and 11 patients in the sustained AF group and three in the paroxysmal AF group were Dabigatran users with *P*-value 0.31,

When we evaluated the baseline characters of patients with HT compared to those without HT, we found that all patients with HT group were statistically significant older with *P*-value < 0.001, statistically significant higher NIHSS at presentation with *P*-value 0.002, statistically significant higher HAS-BLED score with *P*-value 0.008, and higher percentages of patients who were using warfarin with *P*-value < 0.001, we also found that the patients in HI1 and HI2 groups were statistically significant older, with *P*-values < 0.001, < 0.001 respectively, had statistically significant higher NIHSS at presentation with *P*-values 0.004, 0.002 respectively, had statistically significant higher percentages of patients who were using warfarin with *P*-values 0.003, 0.003 respectively, and had statistically significant higher HAS-BLED score with *P*-values 0.01, 0.03 respectively, in addition, the patients in PH type1 and type2 groups were statistically significant older, with *P*-values 0.03, 0.02 respectively, had statistically significant higher percentages of patients with anterior circulation stroke, with *P*-values 0.04, 0.04 respectively, had statistically significant higher NIHSS at presentation with *P*-values 0.002, 0.001 respectively, had statistically significant higher percentages of patients who were using warfarin with *P*-values 0.008, 0.02 respectively, and had statistically significant higher HAS-BLED score with *P*-values 0.03, 0.02 as shown in Table 1.

**Table 1:** Comparison between the non-HT patients and HT patients baseline characters.

Demographic data	Non-HT group N = 509	All patients with HT, N = 207	*P*-value	HI 1, N = 87	*P*-value	HI 2, N = 62	*P*-value	PH 1, N = 33	*P*-value	PH 2, N = 25	*P*-value
Gender, no, (percentage)											
Male, no, (percentage)	299.0 (58.7%)	111.0 (53.6%)	0.21	43.0 (55.2%)	0.10	32.0 (51.6%)	0.28	20.0 (60.6%)	0.83	16.0 (64.0%)	0.60
Age (years) Median (IQR)	65.0 (62.0–73.0)	75.5 (71.5–79.0)	< 0.001*	75.0 (71.5–79.0)	< 0.001*	75.5 (71.5–79.0)	< 0.001*	75.5 (72–79.0)	0.002*	76.0 (72–79.5)	0.003*
Location of infarction, no, (percentage)											
Anterior circulation, no, (percentage)	347.0 (68.2%)	137.0 (66.2%)	0.61	51.0 (58.6%)	0.08	36.0 (58.1%)	0.11	28.0 (84.9%)	0.04*	22.0 (88.0%)	0.04*
NIHSS at time of admission, Median (IQR)	12 (9–17.0)	17.0 (13.0–21.0)	0.002*	16.0 (14.0–21.0)	0.004*	17.0 (13.0–20.0)	0.002*	3.0 (3.0–4.0)	0.03*	3.0 (3.0–4.0)	0.02*
HAS-BLED score, Median (IQR)	1.0 (1.0–2.0)	3.0 (3.0–4.0)	0.008*	3.0 (3.0–4.0)	0.01*	3.0 (3.0–4.0)	0.03*	17.0 (15.0–21.0)	0.002*	17.0 (14.0–21.0)	0.001*
Door to needle time (min.), Median (IQR)	62.0 (55.0–68.0)	60.0 (57.0–70.0)	0.25	60.0 (58.0–68.0)	0.36	60.0 (57.0–68.0)	0.41	61.0 (57.0–70.0)	0.27	61.0 (57.0–69.0)	0.31
Time of receiving IV rtPA from stroke onset (min.), Median (IQR)	178.0 (163.0–200.0)	182.0 (164–208)	0.36	182.0 (164–206)	0.41	181.0 (162–209)	0.48	183.0 (165–208)	0.36	184.0 (164–211)	0.38
Patients on antiplatelets before stroke, no, (percentage)											
Aspirin	52.0 (10.4%)	28.0 (13.5%)	0.52	12.0 (13.8%)	0.80	9.0 (14.5%)	0.75	4.0 (12.1%)	0.75	3.0 (12.0%)	0.46
Clopidogrel	60.0 (12.0%)	24.0 (11.6%)		10.0 (11.5%)		8.0 (12.9%)		4.0 (12.1%)		3.0 (12.0%)	
Ticagrelor	37.0 (7.5%)	18.0 (8.7%)		6.0 (6.9%)		4.0 (6.5%)		3.0 (9.1%)		3.0 (12.0%)	
Patients on anticoagulants before stroke, no, (percentage)											
Warfarin	137.0(26.9%)	93.0 (44.9%)	< 0.001*	37.0 (42.5%)	0.003*	28.0 (45.2%)	0.003*	16.0(48.5%)	0.008*	12.0(48.0%)	0.02*
Rivaroxaban	84.0 (16.5%)	39.0 (18.8%)	0.61	19.0 (21.8%)	0.88	13.0 (21.0%)	0.70	16.0(48.5%)	0.52	12.0 (48.0%)	0.51
Apixaban	76.0 (14.9%)	36.0 (17.4%)		17.0 (19.5%)		10.0 (16.1%)		4.0 (12.1%)		3.0 (12.0%)	
Endoxaban	35.0 (6.9%)	15.0 (7.2%)		6.0 (6.9%)		4.0 (6.5%)		6.0 (18.2%)		3.0 (12.0%)	
Dabigatran	29.0 (5.7%)	14.0 (6.8%)		6.0 (6.9%)		4.0 (6.5%)		3.0 (9.1%)		2.0 (8.0%)	
Patients who underwent endovascular management, no, (percentage)	29.0 (5.7%)	13.0 (6.3%)	0.76	6.0 (6.9%)	0.66	3.0 (4.8%)	0.78	2.0 (6.0%)	0.42	2.0 (6.0%)	0.72

p: *p* value, *: Statistically significant at *p* ≤ 0.05, *HT* hemorrhagic transformation, *HI* hemorrhagic infarction, *PH* parenchymal hematoma, *IQR* inter-quartile range, *HAS-BLED* hypertension, abnormal renal/liver function, stroke, bleeding history or predisposition, labile INR, elderly, drugs/alcohol concomitantly score.

When we evaluated the risk factors of patients with HT compared to those without HT, we found that all patients with HT had statistically significantly higher percentages of patients with sustained AF, with *P*-value < 0.001; we also found that the HI1 and HI2 groups had statistically significantly higher percentages of patients with sustained AF, with *P*-values of 0.001 and 0.02, respectively, in addition, we found that the PH1 and PH2 groups had statistically significantly higher percentages of patients with sustained AF, with *P*-values of 0.002 and 0.003, respectively, as shown in Table 2.

**Table 2:** Comparison between the non-HT patients and HT patients risk factors.

Risk factor, no, (percentage)	Non-HT group N = 509	All HT, N = 207	*P*-value	HI type 1, N = 87	*P*-value	HI type 2, N = 62	*P*-value	PH 1, N = 33	*P*-value	PH 2, N = 25	*P*-value
Hyperlipidemia	140.0 (27.5%)	66.0 (31.9%)	0.24	26.0 (29.9%)	0.65	21.0 (33.9%)	0.29	11.0 (33.3%)	0.78	8.0 (32.0%)	0.70
Diabetes mellitus	144.0 (28.3%)	64.0 (30.9%)	0.66	25.0 (28.7%)	0.93	20.0 (32.3%)	0.51	11.0 (33.3%)	0.95	8.0 (32.0%)	0.93
Hypertension	271.0 (53.2%)	115.0 (55.6%)	0.57	49.0 (56.3%)	0.59	33.0 (53.2%)	0.99	19.0 (57.6%)	0.77	14.0 (56.0%)	0.68
Ischemic heart disease	149.0 (29.7%)	50.0 (24.2%)	0.17	24.0 (27.6%)	0.75	16.0 (25.8%)	0.57	6.0 (18.2%)	0.66	4.0 (16.0%)	0.93
Heart failure	118.0 (23.2%)	42.0 (20.3%)	0.40	20.0 (23.0%)	0.97	13.0 (21.0%)	0.70	5.0 (15.2%)	0.98	4.0 (16.0%)	0.93
Admission hyperglycemia	143.0 (28.1%)	57.0 (27.5%)	0.88	24.0 (27.6%)	0.92	17.0 (27.4%)	0.91	9.0 (27.3%)	0.67	7.0 (28.0%)	0.76
Chronic kidney disease	98.0 (19.3%)	31.0 (15.0%)	0.18	12.0 (13.8%)	0.23	10.0 (16.1%)	0.55	5.0 (15.2%)	0.56	4.0 (16.0%)	0.69
Previous TIA	145.0 (28.5%)	60.0 (29.0%)	0.89	26.0 (29.9%)	0.79	16.0 (25.8%)	0.66	10.0 (30.3%)	0.95	8.0 (32.0%)	0.90
Atrial fibrillation types according to persistence											
Paroxysmal AF	278.0 (54.6%)	70.0 (33.8%)	< 0.001*	31.0 (41.4%)	0.001*	24.0 (38.7%)	0.02*	9.0 (27.3%	0.002*	6.0 (24.0%)	0.003*
Sustained AF	231.0 (45.4%)	137.0 (66.2%)		56.0 (48.3%)		38.0 (61.3%)		24.0 (72.7%)		19.0 (76.0%)	
Co-morbid conditions on admission											
Temperature on admission, Median (IQR)	37.4 (37.2–37.9)	37.6 (37.3–38.1)	0.31	37.6 (37.2–38.1)	0.34	37.6 (37.3–38.1)	0.28	37.6 (37.2–38.1)	0.32	37.6 (37.3–38.1)	0.25
Cholesterol level on admission, mg/dL, Median (IQR)	186.0 (183–249)	194.5 (186.5–253)	0.23	194.0 (187–256)	0.17	192.0 (185–253)	0.26	196.0 (187–246)	0.24	198.0 (185–248)	0.31
Mean value of two points evaluation of BP within 24 h of symptoms onset, mmHg, Median (IQR)	145.0 (130–155)	150.0 (135–160	0.18	150.0 (135–160	0.12	150.0 (135–160	0.21	150.0 (135–160)	0.17	150.0 (135–160)	0.19

p: *p* value, *: Statistically significant at *p* ≤ 0.05, *HT* hemorrhagic transformation, *HI* hemorrhagic infarction, *PH* parenchymal hematoma, *IQR* inter-quartile range, *AF* atrial fibrillation.

We evaluated the predictors of all-HT types and detected that in univariate analysis, sustained AF (*P*-value < 0.001) and warfarin use (*P*-value < 0.001) had statistically significant predictive values of all-HT, also older age at presentation (*P*-value 0.001), baseline NIHSS on admission (*P*-value < 0.001), and HAS-BLED score (*P*-value 0.02) had statistically significant predictive values of all-HT; however, a multivariate regression model showed that older age (odds ratio [OR] 1.14; 95% CI 1.12 to 1.27; *P*-value 0.002), baseline NIHSS score (OR 1.72; 95% CI 1.25 to 1.47; P < 0.001), sustained AF (OR 0.84; 95% CI 0.75 to 0.84; *P*-value 0.003), warfarin use (OR 1.96; 95% CI 1.46 to 1.98; *P*-value 0.001), and HAS-BLED score (OR 1.75; 95% CI 1.35 to 1.83; *P*-value 0.02) independently predict all-HT, as shown in Table 3.

**Table 3:** Univariate and multivariate Logistic regression analysis of the characters and risk factors of all patients with HT (n = 207).

Characters and risk factors	*P*	Univariate OR (LL–UL 95% CI)	*p*	# Multivariate OR (LL–UL 95% CI)
Male	**0.11**	0.98 (0.92–1.41)		
Age at time of presentation	0.001*	1.27* (1.13–1.26)	0.002*	1.14* (1.12–1.27)
Lesion location (Anterior circulation)	**0.09**	0.88 (0.81–1.11)		
NIHSS at time of admission	< 0.001*	1.87* (1.37–1.67)	< 0.001*	1.72* (1.25–1.47)
Admission hyperglycemia	**0.19**	1.02 (0.91–1.42)		
Door to needle time (min)	**0.21**	1.05 (0.87–1.11)		
Time of receiving IV rtPA from stroke onset	**0.27**	0.86 (0.82–1.12)		
Hyperlipidemia	**0.09**	0..76 (0.95–1.11)		
Diabetes mellitus	**0.22**	1.07 (0.91–1.23)		
Hypertension	**0.17**	1.11 (0.95–1.36)		
Previous TIA	**0.11**	0.89 (0.94–1.21)		
Sustained Atrial fibrillation	< 0.001*	1.15* (1.11–1.19)	0.003*	0.84* (0.75–0.84)
Ischemic heart disease	**0.45**	1.23 (0.91–1.19)		
Heart Failure	**0.12**	0.97 (0.87–1.23)		
Chronic kidney disease	**0.38**	0.81 (0.89–1.69)		
Temperature on admission	**0.22**	1.15 (0.78–1.19)		
Cholesterol level on admission	**0.34**	0.91 (0.83–1.32)		
Mean value of two points evaluation of BP within 24 h of symptoms onset	**0.23**	1.14 (0.74–1.38)		
Antiplatelet use	**0.27**	0.98 (0.84–1.09)		
Warfarin use	< 0.001*	1.92 (2.17–2.41)	0.001*	1.96* (1.46–1.98)
NOAC use	**0.20**	1.08 (0.94–1.31)		
HAS-BLED score	0.02*	1.76 (1.36–2.07)	0.02*	1.75* (1.35–1.83)

*OR* Odd’s ratio, *HI* hemorrhagic infarction, *CI* confidence interval, *LL* lower limit, *UL* upper limit, *CA* coronary artery disease, #: All variables with *p* < 0.05 was included in the multivariate, *: Statistically significant at *p* ≤ 0.05, *HAS-BLED* hypertension, abnormal renal/liver function, stroke, bleeding history or predisposition, labile INR, elderly, drugs/alcohol concomitantly score. Significant values are in bold.

We evaluated the predictors of HI 1 and detected that in univariate analysis, sustained AF (*P*-value < 0.001) and warfarin use (*P*-value < 0.001) had statistically significant predictive values of HI 1, also older age at presentation (*P*-value 0.03), baseline NIHSS on admission (*P*-value < 0.001), and HAS-BLED score (*P*-value 0.03) had statistically significant predictive values of HI 1; however, a multivariate regression model showed that older age (odds ratio [OR] 1.05; 95% CI 1.05 to 1.11; *P*-value 0.004), baseline NIHSS score (OR 1.20; 95% CI 1.16 to 1.21; *P* < 0.001), sustained AF (OR 0.77; 95% CI 0.72 to 0.82; *P*-value 0.005), warfarin use (OR 1.41; 95% CI 1.36 to 1.86; *P*-value 0.002), and HAS-BLED score (OR 1.24; 95% CI 1.21 to 1.73; *P*-value 0.04) independently predict HI1, as shown in Table 4.

**Table 4:** Univariate and multivariate Logistic regression analysis of the characters and risk factors of patients with HI 1 (n = 87).

Characters and risk factors	*P*	Univariate OR (LL–UL 95% CI)	*p*	# Multivariate OR (LL–UL 95% CI)
Male	**0.09**	0.97 (0.87–1.36)		
Age at time of presentation	0.003*	1.04* (1.012–1.036)	0.004*	1.05* (1.05–1.11)
Lesion location (Anterior circulation)	**0.08**	0.91 (0.82–1.08)		
NIHSS at time of admission	< 0.001*	1.23* (1.22–1.54)	< 0.001*	1.20* (1.16–1.21)
Admission hyperglycemia	**0.12**	1.04 (0.96–1.31)		
Door to needle time (min)	**0.15**	1.02 (0.94–1.02)		
Time of receiving IV rtPA from stroke onset	**0.23**	0.91 (0.93–1.05)		
Hyperlipidemia	**0.06**	0..86 (0.98–1.13)		
Diabetes mellitus	**0.14**	1.17 (0.93–1.27)		
Hypertension	**0.12**	1.08 (0.97–1.24)		
Previous TIA	**0.07**	1.06 (0.99–1.13)		
Sustained Atrial fibrillation	< 0.001*	1.15* (1.11–1.19)	0.005*	0.77 (0.72–0.82)
Ischemic heart disease	**0.31**	1.05 (0.95–1.16)		
Heart Failure	**0.07**	0.92 (0.84–1.01)		
Chronic kidney disease	**0.43**	0.73 (0.88–1.47)		
Temperature on admission	**0.16**	1.07 (0.83–1.17)		
Cholesterol level on admission	**0.41**	0.87 (0.71–1.21)		
Mean value of two points evaluation of BP within 24 h of symptoms onset	**0.20**	1.08 (0.67–1.34)		
Antiplatelet use	**0.21**	0.96 (0.90–1.02)		
Warfarin use	< 0.001*	1.71 (1.61–1.81)	0.002*	1.41 (1.36–1.86)
NOAC use	**0.11**	1.05 (0.99–1.11)		
HAS-BLED score	0.03*	1.17 (1.12–1.57)	0.04*	1.24 (1.21–1.73)

*OR* odd’s ratio, *HI* hemorrhagic infarction, *CI* confidence interval, *LL* lower limit, *UL* upper limit; *CAD* coronary artery disease, #: All variables with *p* < 0.05 was included in the multivariate.

*: Statistically significant at *p* ≤ 0.05, *HAS-BLED* hypertension, abnormal renal/liver function, stroke, bleeding history or predisposition, labile INR, elderly, drugs/alcohol concomitantly score. Significant values are in bold.

We evaluated the predictors of the occurrence of HI 2. The univariate analysis showed that sustained AF (*P*-value < 0.001) and warfarin use (*P*-value < 0.001) had statistically significant predictive values of HI 2, also older age at presentation (*P*-value 0.002), baseline NIHSS on admission (*P*-value < 0.001), and HAS-BLED score (*P*-value 0.02) had a predictive value of HI 2; however, a multivariate regression model showed that sustained AF (OR 0.67; 95% CI 0.69 to 0.87; *P*-value 0.003), warfarin use (OR 1.41; 95% CI 1.36 to 1.86; *P*-value 0.002), older age (OR 1.09; 95% CI 1.03 to 1.34; *P*-value 0.03), baseline NIHSS score (OR 1.41; 95% CI 1.06 to 2.11; P < 0.001), and HAS-BLED score (OR 1.36; 95% CI 1.16 to 1.67; *P*-value 0.04) independently predict HI 2, as shown in Table 5.

**Table 5:** Univariate and multivariate Logistic regression analysis of the characters and risk factors of patients with HI 2 (n = 62).

Characters and risk factors	*P*	Univariate OR (LL–UL 95% CI)	*p*	# Multivariate OR (LL–UL 95% CI)
Male	**0.08**	1.23 (0.93–1.36)		
Age at time of presentation	0.002*	1.58* (1.04–1.13)	0.03*	1.05* (1.03–1.34)
Lesion location (Anterior circulation)	**0.11**	1.12 (0.98–1.07)		
NIHSS at time of admission	< 0.001*	1.82* (1.14–1.93)	< 0.001*	1.41* (1.06–2.11)
Admission hyperglycemia	**0.23**	1.04 (0.96–1.31)		
Door to needle time (min)	**0.18**	1.09 (0.89–1.09)		
Time of receiving IV rtPA from stroke onset	**0.17**	0.87 (0.84–1.11)		
Hyperlipidemia	**0.09**	1.02 (0.97–1.08)		
Diabetes mellitus	**0.29**	1.13 (0.82–1.09)		
Hypertension	**0.31**	0.98 (0.96– 1.34)		
Previous TIA	**0.09**	1.17 (0.97–1.27)		
Sustained Atrial fibrillation	< 0.001*	1.24* (1.08–1.27)	0.003*	0.67 (0.69–0.87)
Ischemic heart disease	**0.28**	0.98 (0.81–1.09)		
Heart Failure	**0.11**	0.92 (0.84–1.01)		
Chronic kidney disease	**0.47**	0.69 (0.76–1.74)		
Temperature on admission	**0.33**	1.07 (0.97–1.27)		
Cholesterol level on admission	**0.27**	0.87 (0.96–1.31)		
Mean value of two points evaluation of BP within 24 h of symptoms onset	**0.21**	1.06 (0.79–1.24)		
Antiplatelet use	**0.21**	0.96 (0.90–1.02)		
Warfarin use	< 0.001*	1.71 (1.61–1.81)	0.002*	1.41 (1.36–1.86)
NOAC use	**0.17**	1.05 (0.99–1.11)		
HAS-BLED score	0.02*	1.43 (1.24–2.13)	0.04*	1.36 (1.19–1.67)

*OR* odd’s ratio, *HI* hemorrhagic infarction, *CI* confidence interval, *LL* lower limit, *UL* upper limit, *CAD* coronary artery disease, #: All variables with *p* < 0.05 was included in the multivariate.

*: Statistically significant at *p* ≤ 0.05, *HAS-BLED* hypertension, abnormal renal/liver function, stroke, bleeding history or predisposition, labile INR, elderly, drugs/alcohol concomitantly score. Significant values are in bold.

We evaluated the contribution of the AF type and treatment in the occurrence of PH 1; we detected that in univariate analysis, sustained AF (*P*-value 0.002) and warfarin use (*P*-value 0.002) had statistically significant predictive values of PH 1, also older age at presentation (*P*-value 0.009), baseline NIHSS on admission (*P*-value < 0.001), anterior circulation stroke (*P*-value 0.01), and HAS-BLED score (*P*-value 0.03) had statistically significant predictive values of PH 1; however, a multivariate regression model showed that older age (odds ratio [OR] 1.27; 95% CI 1.18 to 1.83; *P*-value 0.04), baseline NIHSS score (OR 1.40; 95% CI 1.55 to 1.81; P < 0.001), anterior-circulation stroke (OR 1.53; 95% CI 1.29 to 1.67; *P*-value 0.04), sustained AF (OR 1.16; 95% CI 1.23 to 1.47; *P*-value 0.005), warfarin use (OR 1.42; 95% CI 1.46 to 1.92; *P*-value 0.03), and HAS-BLED score (OR 1.23; 95% CI 1.27 to 2.04; *P*-value 0.05) independently predict PH 1, as shown in Table 6.

**Table 6:** Univariate and multivariate Logistic regression analysis of the characters and risk factors of patients with PH 1 (n = 33).

Characters and risk factors	*P*	Univariate OR (LL–UL 95% CI)	*p*	# Multivariate OR (LL–UL 95%C.I)
Male	**0.21**	1.12 (0.91–1.34)		
Age at time of presentation	0.009*	1.24* (1.31–1.87)	0.04*	1.27* (1.18–1.83)
Lesion location (Anterior circulation)	0.01*	1.09* (1.11–1.58)	0.04*	1.53* (1.29–1.67)
NIHSS at time of admission	< 0.001*	1.31* (1.26–1.33)	< 0.001*	1.40* (1.55–1.81)
Admission hyperglycemia	**0.23**	1.17 (0.89–1.09)		
Door to needle time (min)	**0.11**	0.93 (0.94–1.21)		
Time of receiving IV rtPA from stroke onset	**0.27**	0.82 (0.85–1.34)		
Hyperlipidemia	**0.31**	1.43 (0.93–1.74)		
Diabetes mellitus	**0.21**	1.23 (0.92–1.64)		
Hypertension	**0.17**	0.98 (0.97–1.78)		
Previous TIA	**0.11**	0.92 (0.93–1.29)		
Ischemic heart disease	**0.36**	0.74 (0.71–1.56)		
Sustained Atrial fibrillation	0.002*	1.26* (1.14–1.79)	0.005*	1.16 (1.23–1.47)
Heart Failure	**0.18**	0.87 (0.79–1.06)		
Chronic kidney disease	**0.41**	0.93 (0.86–1.29)		
Admission hyperglycemia	**0.21**	1.13 (0.86–1.39)		
Temperature on admission	**0.18**	0.83 (0.87–1.45)		
Cholesterol level on admission	**0.31**	1.34 (0.92–1.09)		
Mean value of two points evaluation of BP within 24 h of symptoms onset	**0.29**	1.41 (0.76–1.53)		
Antiplatelet use	**0.21**	0.96 (0.90–1.02)		
Warfarin use	0.002*	1.31 (1.61–1.75)	0.03*	1.42 (1.46–1.92)
NOAC use	**0.34**	1.07 (0.96–1.21)		
HAS-BLED score	0.03*	1.19 (1.32–1.94)	0.05*	1.23 (1.27–2.04)

*OR* odd’s ratio, *PH* parenchymal hematoma, *CI* confidence interval, *LL* lower limit, *UL* upper limit, *CAD* coronary artery disease, #: All variables with *p* < 0.05 was included in the multivariate.

*: Statistically significant at *p* ≤ 0.05, *HAS-BLED* hypertension, abnormal renal/liver function, stroke, bleeding history or predisposition, labile INR, elderly, drugs/alcohol concomitantly score. Significant values are in bold.

We evaluated the contribution of the AF type and treatment in the occurrence of PH 2, we detected that in univariate analysis, sustained AF (*P*-value 0.004) and warfarin use (*P*-value 0.04) had statistically significant predictive values of PH 2, also older age at presentation (*P*-value 0.007), baseline NIHSS on admission (*P*-value 0.003), anterior-circulation stroke (*P*-value 0.02), and HAS-BLED score (*P*-value 0.03) had statistically significant predictive values of PH 2; however, a multivariate regression model showed that older age (odds ratio [OR] 1.23; 95% CI 1.09 to 1.62; *P*-value 0.02), baseline NIHSS score (OR 1.40; 95% CI 1.49 to 1.92; *P*-value 0.006), anterior-circulation stroke (OR 1.09; 95% CI 1.18 to 1.54; *P*-value 0.04), sustained AF (OR 1.64; 95% CI 1.16 to 1.77; *P*-value 0.007), warfarin use (OR 1.35; 95% CI 1.26 to 1.97; *P*-value 0.04), and HAS-BLED score (OR 1.12; 95% CI 1.17 to 1.59; *P*-value 0.04) independently predict PH 2, as shown in Table 7.

**Table 7:** Univariate and multivariate Logistic regression analysis of the characters and risk factors of patients with PH 2 (n = 25).

Characters and risk factors	*P*	Univariate OR (LL–UL 95% CI)	*p*	# Multivariate OR (LL–UL 95%C.I)
Male	**0.37**	1.36 (0.78–1.27)		
Age at time of presentation	0.007*	1.18* (1.29–1.76)	0.02*	1.23* (1.09–1.62)
Lesion location (Anterior circulation)	0.02*	0.98* (1.23–1.57)	0.04*	1.09* (1.18–1.54)
NIHSS at time of admission	0.003*	1.27* (1.34–1.83)	0.006*	1.40* (1.49–1.92)
Admission hyperglycemia	**0.18**	1.23 (0.86–1.27)		
Door to needle time (min)	**0.34**	0.91 (0.92–1.38)		
Time of receiving IV rtPA from stroke onset	**0.28**	1.31 (0.82–1.14)		
Hyperlipidemia	**0.43**	1.27 (0.91–1.69)		
Diabetes mellitus	**0.35**	1.17 (0.84–1.56)		
Hypertension	**0.19**	0.95 (0.92—1.67)		
Previous TIA	**0.23**	0.92 (0.83–1.38)		
Ischemic heart disease	**0.33**	0.79 (0.78—1.76)		
Sustained Atrial fibrillation	0.004*	1.26* (1.14–1.79)	0.007*	1.64 (1.16–1.77)
Heart Failure	**0.16**	0.85 (0.84–1.19)		
Chronic kidney disease	**0.38**	1.19 (0.91–1.37)		
Admission hyperglycemia	**0.32**	1.26 (0.89–1.49)		
Temperature on admission	**0.28**	0.78 (0.83–1.65)		
Cholesterol level on admission	**0.43**	1.14 (0.91–1.23)		
Mean value of two points evaluation of BP within 24 h of symptoms onset	**0.37**	1.31 (0.81–1.43)		
Antiplatelet use	**0.27**	0.83 (0.90–1.17)		
Warfarin use	0.007*	1.41 (1.81–2.08)	0.04*	1.35 (1.26–1.97)
NOAC use	**0.37**	1.17 (0.92–1.39)		
HAS-BLED score	0.04*	1.08 (1.13–1.68)	0.04*	1.12 (1.17–1.59)

*OR* odd’s ratio, *PH* parenchymal hematoma, *CI* confidence interval, *LL* lower limit, *UL* upper limit, *CAD* coronary artery disease, #: All variables with *p* < 0.05 was included in the multivariate.

*: Statistically significant at *p* ≤ 0.05, *HAS-BLED* hypertension, abnormal renal/liver function, stroke, bleeding history or predisposition, labile INR, elderly, drugs/alcohol concomitantly score. Significant values are in bold.

## Discussion

Many studies showed that stroke caused by AF was associated with an increased risk of post-alteplase HT, functional disability, and mortality^6–8^. We classified HT according to ECASS as HT is not one entity and its subtypes are very different in their incidence and contribution to post-HT mortality and functional dependence, making HI 1 the most common type and the PH 2 the most dangerous type^28^. So, we aim to investigate predictors of HT in general and ECASS-based HT subtypes.

Our study differed from other studies that evaluated post-alteplase HT as it is the first-ever prospective study that assessed the ability of different factors, including types and treatment of AF, to predict ECASS-based subtypes of post-alteplase hemorrhagic transformation in AF patients presenting with embolic stroke and administered alteplase.

We determined our primary endpoint as the predictors of all HT in AF patients presenting with ischemic stroke. Our secondary endpoints were the predictors of HI 1, HI 2, PH 1 and PH 2.

In our study, 28.9% of the patients had a haemorrhagic transformation, which agrees with the findings of Strbian et al. and Sun et al.^15,29^, who stated that up to 30% of AIS patients who were treated with alteplase had HT, and 118 participants (16.5%) had symptomatic worsening due to HT.

We found that sustained AF and warfarin use were independent predictors of all ECASS-based post-alteplase HT subtypes. However, there was no such study that evaluated the ability of AF types or treatment to predict post-alteplase HT; our findings agreed partially with the conclusions from Steinberg et al., who found that sustained AF was associated with a higher percentage of hemorrhagic stroke than paroxysmal AF in patients who did not receive alteplase^30^; also our results agreed with the findings of ROCKET AF and ARISTOTLE studies which found that using warfarin was associated with a higher incidence of intracranial hemorrhage compared with direct oral anticoagulants in embolic stroke patients^9,10^.

The higher incidence of hemorrhagic complications associated with sustained AF might be explained as patients who experienced sustained AF usually used anticoagulants for a longer duration than paroxysmal AF, which makes them more liable to an increased incidence of hemorrhagic complications. Also, sustained AF patients usually experience more prominent and severe electromechanical and hemodynamic sequelae of the prolonged irregular rhythm, which worsens the outcomes of any embolic events^30^. Moreover, many studies showed that the longer the duration of the AF the worse the clinical outcomes of the AF-related thromboembolic events^31^. The (ASSERT) study suggested that as little as six minutes of AF increased stroke risk^32^, while Capucci et al.^33^ reported that AF lasting ≥ 24 h led to three times rise in thromboembolic risk and poor outcomes compared to those without AF episodes lasting for 24 h.

Our study showed that older age was an independent predictor of different subtypes of post-alteplase HT. These findings aligned with those of Liu et al. and Sun et al.^15,34^, who found that age ≥ 68 and age ≥ 70, respectively, were predictors of haemorrhagic transformation in AIS patients treated with alteplase.

Also, we found that higher baseline NIHSS was an independent predictor of all Haemorrhagic infarction subtypes. At the same time, anterior-circulation stroke was an independent predictor of PH 1 and PH 2, which agreed with Chenna et al., Dornak et al., Sun et al., and Xue et al.^15,35–37^.

Moreover, we detected that the HAS-BLED score was an independent predictor of different subtypes of post-alteplase HT; this finding was partially in line with the findings of Gallego et al. and Chan et al. who found that the HAS-BLED score was an independent predictor of haemorrhagic complications in anticoagulated atrial fibrillation patients^20,38^.

Although our study was the first-ever prospective one that evaluated the predictors of post-alteplase HT subtypes in AF patients presenting with first-ever embolic stroke, it had some disadvantages. First, our study was prospective, so the included population was relatively small; second, our participants were primarily from Egypt and the United Arab Emirates, which limited the generalization of our findings; third, we could not include some factors in our analysis such as the white matter lesions burden as we did not have the needed MRI software program, so we need a multi-centre study included patients from different ethnicities to allow generalization of the results.

## Conclusion

In atrial fibrillation patients presented with first-ever embolic stroke and treated with alteplase in Egypt and the United Arab Emirates, older age, higher NIHSS, sustained AF, warfarin use, and higher HAS-BLED score were independent predictors of all ECASS-based subtypes of haemorrhagic infarction; in addition, anterior-circulation stroke was an independent predictor of PH 1 and PH 2.

## Data Availability

The datasets generated and analyzed during the current study are not publicly available due to the ethical regulations of our university, but are available from the corresponding author (Mohamed G. Zeinhom) on reasonable request.

## Abbreviations

AIS: Acute ischemic stroke
ACS: Anterior-circulation stroke
CNS: Central nervous system
CT: Computerized tomography
ECASS: European cooperative acute stroke study
ECG: Electrocardiogram
FDA: Food and Drug Administration
HDL: High density lipoprotein
HI1: Hemorrhagic infarction type1
HI2: Hemorrhagic infarction type2
HT: Hemorrhagic transformation
INR: International normalization ratio
IQR: Inter quartile range
LDL: Low density lipoprotein
MRA: Magnetic resonance angiography
MRI: Magnetic resonance imaging
mRS: Modified Rankin scale
NIHSS: National institute of health stroke score
PCS: Posterior-circulation stroke
PH1: Parenchymal hematoma type 1
PH2: Parenchymal hematoma type 2
TPA: Recombinant tissue plasminogen activator
TIA: Transient ischemic attack
